# Knowledge of heart attack and stroke symptomology: a cross-sectional comparison of rural and non-rural US adults

**DOI:** 10.1186/1471-2458-12-283

**Published:** 2012-06-01

**Authors:** Michael T Swanoski, May Nawal Lutfiyya, Maria L Amaro, Michael F Akers, Krista L Huot

**Affiliations:** 1 College of Pharmacy, Ambulatory Care Residency Program, University of Minnesota, Minneapolis, MN, 55455, USA; 2 Essentia Institute of Rural Health, Duluth, MN, 55803, USA; 3 Essentia Health System, Ambulatory Care Pharmacy Services, Duluth, MN, 55805, USA

**Keywords:** Rural public health, Heart attack symptoms, Stroke symptoms, Knowledge of heart attack and stroke symptomology

## Abstract

**Background:**

Understanding the signs and symptoms of heart attacks and strokes are important not only in saving lives, but also in preserving quality of life. Findings from recent research have yielded that the prevalence of cardiovascular disease risk factors are higher in rural populations, suggesting that adults living in rural locales may be at higher risk for heart attack and/or stroke. Knowledge of heart attack and stroke symptomology as well as calling 911 for a suspected heart attack or stroke are essential first steps in seeking care. This study sought to examine the knowledge of heart attack and stroke symptoms among rural adults in comparison to non-rural adults living in the U.S.

**Methods:**

Using multivariate techniques, a cross-sectional analysis of an amalgamated multi-year Behavioral Risk Factor Surveillance Survey (BRFSS) database was performed. The dependent variable for this analysis was low heart attack and stroke knowledge score. The covariates for the analysis were: age, sex, race/ethnicity, annual household income, attained education, health insurance status, having a health care provider (HCP), timing of last routine medical check-up, medical care deferment because of cost, self-defined health status and geographic locale.

**Results:**

The weighted n for this study overall was 103,262,115 U.S. adults > =18 years of age. Approximately 22.0% of these respondents were U.S. adults living in rural locales. Logistic regression analysis revealed that those U.S. adults who had low composite heart attack and stroke knowledge scores were more likely to be rural (OR = 1.218 95%CI 1.216-1.219) rather than non-rural residents. Furthermore, those with low scores were more likely to be: male (OR = 1.353 95%CI 1.352-1.354), >65 years of age (OR = 1.369 95%CI 1.368-1.371), African American (OR = 1.892 95%CI 1.889-1.894), not educated beyond high school (OR = 1.400 955CI 1.399-1.402), uninsured (OR = 1.308 95%CI 1.3-6-1.310), without a HCP (OR = 1.216 95%CI 1.215-1.218), and living in a household with an annual income of < $50,000 (OR = 1.429 95%CI 1.428-1.431).

**Conclusions:**

Analysis identified clear disparities between the knowledge levels U.S. adults have regarding heart attack and stroke symptoms. These disparities should guide educational endeavors focusing on improving knowledge of heart attack and stroke symptoms.

## Background

In the United States, the total cost(s) of cardiovascular disease (CVD) in 2010 were estimated to be $444 billion [1]. Approximately one of every six dollars spent on health care in this country was for the treatment of CVD. Since CVD is age-prevalent, the economic impact on the U.S. health care system will become even greater as the population ages [1]. CVD encompasses numerous disease states, with heart attacks and strokes being among the most notable. For instance, in the U.S., heart disease is the number one cause of death [2] while stroke is the leading cause of long-term adult disability [3]. Therefore, early treatment for both heart attack and stroke is critical in lowering the risk for poor outcomes [4]. Understanding the signs and symptoms of heart attacks and strokes are important not only in saving lives, but also in preserving quality of life [5,6].

Recent research has yielded that the prevalence of cardiovascular disease risk factors are higher in rural populations [7-11] suggesting that those adults may be at higher risk for heart attack and/or stroke [12]. Knowledge of heart attack and stroke symptomology as well as calling 911 as the appropriate first response to suspected heart attack or stroke are essential first steps in seeking care [6].

A number of studies have been conducted in order to ascertain specific populations of adults with knowledge deficits in the areas of first responder action as well as heart attack and stroke symptomology. By identifying these populations of adults, better public health campaigns can be aimed at enhancing knowledge [13-21]. Despite these efforts, there has been very little research conducted to examine the knowledge level of rural U.S. populations regarding stroke and heart attack symptomology. In 2004, a small regional study encompassing the populations of two rural counties in Montana was conducted to assess the knowledge of stroke symptoms in rural adults aged 45 years and older [22]. It was found that study participants were generally aware of stroke symptomology, but not necessarily of stroke risk factors.

An epidemiological gap regarding the knowledge of heart attack and stroke symptoms among residents in rural U.S. communities is evident. This study sought to fill this knowledge gap by examining the level of understanding of heart attack and stroke symptoms among rural adults in comparison to non-rural adults living in the U.S.

## Methods

Bivariate and multivariate techniques were used to analyze a multiyear Behavioral Risk Factor Surveillance Survey (BRFSS) heart attack and stroke module database. BRFSS data are collected using a random-digit dial telephone survey targeting adults 18–99 years of age. These data are collected under the guidance of the Centers for Disease Control and Prevention (CDC) in collaboration with all U.S. states and most U.S. territories. Once collected, BRFSS data are weighted such that they are representative of the non-institutional U.S. population by surveyed state. The data are cross-sectional and are focused on health risk factors and behaviors. A detailed description of the survey design and sampling measures can be found elsewhere [23].

Data from the BRFSS optional module on heart attack and stroke were used in these analyses. Because different states use this module in different years, we merged 2005, 2007, and 2009 data to include as many states and territories as possible. In 2005, 14 states, the District of Columbia and United States Virgin Islands (USVI) included a module in their BRFSS surveys regarding symptoms of heart attack and stroke. In 2007, 13 states, the District of Columbia and the USVI included the module. In 2009, it was included by 19 states and the District of Columbia. Data from 25 states, the USVI, and the District of Columbia were used in these analyses. If a state used the module more than once, only the data from the most recent year were used. For the years in question, the BRFSS heart and stroke module included 13 questions focused on ascertaining knowledge of early symptoms of heart attack and stroke. Of these 13 questions, six were on knowledge of stroke symptoms, six were on knowledge of heart attack symptoms, and one question was on proper first response to either stroke or heart attack.

Respondents were asked if the following were warning signs of stroke: sudden confusion; trouble speaking or understanding; sudden numbness or weakness of face, arm, or leg; sudden trouble seeing in one or both eyes; sudden trouble walking, dizziness, or loss of balance or coordination; or sudden, severe headache with no known cause. An incorrect sign (i.e., sudden chest pain) was included to examine the possibility that respondents would answer “yes” for all the symptoms. Likewise, respondents were asked if the following were warning signs of a heart attack: pain or discomfort in the jaw, neck, or back; feeling weak, lightheaded, or faint; chest pain or discomfort; pain or discomfort in the arms or shoulders; shortness of breath. As was the case with stroke symptoms, an incorrect sign (i.e., trouble seeing in one or both eyes) was included to examine the possibility that respondents would answer “yes” for all the symptoms.

We chose to group the questions for heart attack and stroke symptomology together for analysis because these disorders are both vascular events that require the need for prompt recognition of symptoms and pre-hospital action by either the patient or bystanders. Any costly public health campaign will likely need to address both these vascular diseases together, and strokes are often referred to as “brain attacks,” as many aspects of early stroke management mimic heart attack management [24,25].

For analysis we computed a Heart Attack and Stroke Knowledge Score for each respondent. Correct answers received one point and were categorized according to the following scale: low score 0–5 points, midrange score 6–9 points and high score 10–13 points. Although this scale, like most, is somewhat arbitrary, we based the cut points on the actual range (0 – 13) derived from responses. This scale served the purpose of allowing for the standardized comparison of knowledge levels.

The covariates for the analysis were: sex, age, race/ethnicity, annual household income, education attained, marital status, geographic locale, timing of last routine medical checkup, having a personal health care provider (HCP), having health insurance, deferment of medical care because of cost, and self-defined health status.

The Metropolitan Statistical Area (MSA) variable included in BRFSS was used to define geographic locale and was recoded into the dichotomous categories of rural or non-rural. Rural residents were defined as people living either within an MSA that had no center city or outside an MSA. Non-rural residents included all respondents living in a center city of an MSA, outside the center city of an MSA but inside the county containing the center city, or inside a suburban county of an MSA.

Race and ethnicity was also a computed variable calculated from participant responses to two separate survey questions—one regarding race and the other regarding Latino/Hispanic ethnicity. Combining the responses to these two questions allowed for the derivation of the race and ethnicity variable used in the analyses. All race/ethnicity categories were computed as mutually exclusive entities. For example, all respondents coded as Caucasian chose White as their racial classification, likewise black for African American, etc. If a respondent identified themselves as Hispanic, they were classified by that ethnic category regardless of any additional racial classification. The category of Other/Multi-racial was also calculated. All racial categories were non-Hispanic.

A number of additional original BRFSS variables—age, education attained, marital status, and annual household income—were recoded for these analyses. Age was recoded from a continuous variable to a categorical one with three factors/levels (18–44 years, 45–64 years, and > =65 years). For education, marital status and household income, recoding entailed the collapsing of multiple response categories into fewer ones—three for education and two factors each for household income and marital status. The three factors for education were less than high school, at least high school, and university graduate. Annual household income was categorized into the categories of < $50,000 and > $50,000. Marital status was collapsed into the categories of married or living with a partner and not married or living with a partner. Three health care access variables: having a health care provider, deferment of medical care because of cost, and health insurance status were also included in these analyses. The response categories of “don’t know” and “refused to answer” were recoded as missing data and removed from the analyses.

Bivariate analysis describing the three-level composite scores by each covariate was conducted as well as a description of correct and incorrect answers for each heart attack and stroke symptomology knowledge question by geographic locale (non-rural, rural and total U.S.).

Two logistic regression models were performed using low scores on the combined heart attack and stroke knowledge questions as the dependent variable. All U.S. adults were included in the first model and only rural adults > 18 years of age responding to the survey questions were included in the second. The covariates entered into the two models were: sex, age, race/ethnicity, education attained, marital status, annual household income, geographic locale (first model only), health status, having a personal HCP, health insurance status in the past 12 months, deferring medical care in the past 12 months because of cost, and timing of last routine medical check-up. Alpha was set at 0.05 for all tests of statistical significance. To further reduce bias, a constant was entered into the models. SPSS version 19.0 (SPSS, IBM, Chicago, IL) was used to complete the analyses to account for the complex survey design. Human subjects’ approval was sought and received from Essentia Health’s Institutional Review Board (IRB).

## Results

From the multi-year amalgamated database complied for this study, 103,262,115 U.S. adults > =18 years of age from 25 states, the USVI and the District of Columbia responded to the optional heart attack and stroke symptom module. Approximately 22.0% of these respondents were U.S. adults living in rural locales. Table 1 describes the population responding to the heart attack and stroke symptom module questions in 2005, 2007 and 2009.

**Table 1:** Description of U.S. Adults Responding to Heart Attack and Stroke Symptomology Survey Questions 2005, 2007 and 2009 BRFSS Data (weighted n = 103,262,115)

**Covariates and Factors**		**Percent**
Sex	Male	48.6
	Female	51.4
Age	18-44 Years Of Age	49.8
	45-64 Years Of Age	33.1
	> = 65 Years Of Age	17.1
Race And Ethnicity	Caucasian	72.0
	African American	10.9
	Hispanic	10.2
	Other/Multiracial	6.9
Education Attained	Did Not Graduate From HS	10.9
	HS Graduate	56.3
	University Graduate	32.8
Marital Status	Married Or Living With Partner	64.1
	Not Married Or Living With Partner	35.9
Annual Household Income	<$50,000	53.1
	> = $50,000	46.9
Geographic Locale	Non-Rural	78.1
	Rural	21.9
Health Status	Good To Excellent Health	83.9
	Fair To Poor Health	16.1
Health Insurance Status	Have Health Insurance	85.2
	Do Not Have Health Insurance	14.8
Have Health Care Provider	Have HCP	80.8
	Do Not Have HCP	19.2
Deferment of Medical Care	Medical Care Deferred Because of Cost	13.8
	Medical Care Not Deferred	86.2
Timing of Routine Medical Check Up	Within Last 12 Months	67.3
	Longer Than 12 Months Ago	32.7

Table 2 displays the bivariate analysis of the composite heart attack and stroke three-level knowledge score by the study covariates. Notably this analysis yielded that higher percentages of males as well as those adults defining their health as fair to poor, without health insurance, without a personal HCP, deferring medical care because of cost, having their last routine medical check-up longer than 12 months ago, living in households with an annual income of < $50,000, who are Hispanic, and living in rural locales had a low composite heart attack and stroke symptom knowledge score. This analysis also revealed that not having graduated from high school was a strong bivariate predictor of having a low composite heart attack and stroke symptomology knowledge score.

**Table 2:** Composite Heart Attack and Stroke Symptomology Knowledge Score By Covariates 2005, 2007 and 2009 BRFSS Data

**Covariates and Factors**		**Three Level Composite Score**		
		% Low Composite Score*	% Mid-Range Composite Score*	% High Composite Score*
Sex	Male	8.6	36.9	54.5
	Female	6.3	31.7	62.0
Health Status	Good To Excellent Health	6.6	33.4	60.0
	Fair To Poor Health	11.2	38.4	50.5
Health Insurance Status	Have Health Insurance	6.3	32.6	61.1
	Do Not Have Health Insurance	13.1	42.7	44.1
Have Health Care Provider	Have HCP	6.3	32.6	61.0
	Do Not Have HCP	11.6	40.8	47.7
Medical Care Deferred	Deferred Because Of Cost	9.6	39.5	50.9
	Care Not Deferred	6.9	33.3	59.8
Routine Medical Check Up	Within Last 12 Months	7.1	33.0	59.9
	Longer Than 12 Months Ago	8.0	37.0	55.0
Education Attained	Did Not Graduate From HS	20.9	46.1	33.0
	HS Graduate	7.2	36.6	56.2
	University Graduate	3.3	26.4	70.3
Household Income	<$50,000	9.6	38.7	51.7
	> = $50,000	3.1	27.4	69.5
Race And Ethnicity	Caucasian	5.0	31.5	63.5
	African American	12.4	42.7	44.9
	Hispanic	20.7	43.7	35.6
	Other/Multiracial	10.7	39.5	49.8
Geographic Locale	Non-Rural	7.3	34.2	58.6
	Rural	7.7	34.5	57.8

*By z-statistic column proportions differ significantly from each other at the .05 level.

A bivariate analysis of the percent correct and incorrect responses by symptom question and geographic locale of respondents (non-rural, rural and total U.S.) is displayed in Table 3. For nine of the 13 questions (69.3%) on heart attack and stroke symptomology (three heart attack questions, four stroke questions, and appropriate first response question), rural respondents had higher percentages of *incorrect* answers.

**Table 3:** **Heart Attack and Stroke Symptomology Knowledge Questions With**% **Incorrect and Correct Responses U.S. Adults by Geographic Locale 2005, 2007 and 2009 behavioral risk factor surveillance data**

**Survey Question (Correct Response)**		**Geographic Locale**		**Total U.S.**
		**Non-Rural**	**Rural**	
Do you think pain or discomfort in the jaw, neck, or back are symptoms of a heart attack? (Yes)	Incorrect Answer	46.6	44.2	46.0
	Correct Answer	53.4	55.8	54.0
Do you think feeling weak, lightheaded, or faint are symptoms of a heart attack? (Yes)	Incorrect Answer	36.7	37.2	36.8
	Correct Answer	63.3	62.8	63.2
Do you think chest pain or discomfort are symptoms for a heart attack? (Yes)	Incorrect Answer	7.2	7.7	7.3
	Correct Answer	92.8	92.3	92.7
Do you think sudden trouble seeing in one or both eyes is a symptom of a heart attack? (No)	Incorrect Answer	57.3	59.2	57.8
	Correct Answer	42.7	40.8	42.2
Do you think pain or discomfort in the arms or shoulders are symptoms of a heart attack? (Yes)	Incorrect Answer	13.5	13.5	13.5
	Correct Answer	86.5	86.5	86.5
Do you think shortness of breath is a symptom of a heart attack? (Yes)	Incorrect Answer	15.1	15.0	15.1
	Correct Answer	84.9	85.0	84.9
Do you think sudden confusion or trouble speaking are symptoms of a stroke? (Yes)	Incorrect Answer	10.2	10.7	10.3
	Correct Answer	89.8	89.3	89.7
Do you think sudden numbness or weakness of face, arm, or leg, especially on one side are symptoms of a stroke? (Yes)	Incorrect Answer	6.1	6.3	6.2
	Correct Answer	93.9	93.7	93.8
Do you think sudden trouble seeing in one or both eyes is a symptom of a stroke? (Yes)	Incorrect Answer	27.6	29.7	28.1
	Correct Answer	72.4	70.3	71.9
Do you think sudden chest pain or discomfort are symptoms of a stroke? (No)	Incorrect Answer	58.7	62.6	59.7
	Correct Answer	41.3	37.4	40.3
Do you think sudden trouble walking, dizziness, or loss of balance are symptoms of a stroke? (Yes)	Incorrect Answer	14.3	15.2	14.6
	Correct Answer	85.7	84.8	85.4
Do you think severe headache with no known cause is a symptom of a stroke? (Yes)	Incorrect Answer	39.1	38.4	38.9
	Correct Answer	60.9	61.6	61.1
If you thought someone was having a heart attack or a stroke, what is the first thing you would do? (call 911)	Incorrect Answer	12.1	16.1	13.1
	Correct Answer	87.9	83.9	86.9

Table 4 displays the results of the two logistic regression models performed. The first logistic regression analysis using low composite heart attack and stroke symptom knowledge score as the dependent variable revealed that those US adults who had low composite heart attack and stroke knowledge scores were more likely to be rural (OR = 1.218 95%CI 1.216-1.219) rather than non-rural residents. Furthermore, those with low scores were more likely to be: male (OR = 1.353 95%CI 1.352-1.354), >65 years of age (OR = 1.369 95%CI 1.368-1.371), African American (OR = 1.892 95%CI 1.889-1.894), not educated beyond high school (OR = 1.400 955CI 1.399-1.402), uninsured (OR = 1.308 95%CI 1.3-6-1.310), without a HCP (OR = 1.216 95%CI 1.215-1.218), and living in a household with < $50,000 annual income(OR = 1.429 95%CI 1.428-1.431).

**Table 4:** Logistic Regression Model of Low Composite Heart Attack and Stroke Knowledge Scorers 2005, 2007 and 2009 Amalgamated BRFSS Database

**Covariate**	**Factor**	**Geographic Locale**	
		**US Total Adjusted Odds Ratio (95% CI)**	**Rural Adjusted Odds Ratio (95% CI)**
**Sex**	Male	1.353 (1.352, 1.354)	1.457(1.454,1.459)
	Female	--*	--*
**Age**	18-44 years of age	--*	--*
	45-64 years of age	.811 (.810, .812)	.771 (.770,.773)
	>65 years of age	1.369 (1.368, 1.371)	1.269 (1.265,1.272)
**Race and Ethnicity**	Caucasian	--*	--*
	African American	1.892 (1.889, 1.894)	1.763 (1.758,1.768)
	Hispanic	.821 (.819, .822)	2.787 (2.772,2.802)
	Other/Multiracial	1.041 (1.039, 1.043)	1.487 (1.481,1.493)
**Education Attained**	Did Not Graduate From High School	2.021 (2.018, 2.024)	3.177 (3.166,3.188)
	High School Graduate	1.400 (1.399, 1.402)	1.688 (1.684,1.692)
	University Graduate	--*	--*
**Marital Status**	Married Or Living With Partner	--*	--*
	Not Married Or Living With Partner	1.080 (1.079, 1.082)	1.149 (1.147,1.152)
**Annual Household Income**	<$50,000	1.429 (1.428, 1.431)	1.438 (1.434,1.441)
	>$50,000	--*	--*
**Geographic Locale**	Non-Rural	--*	
	Rural	1.218 (1.216, 1.219)	
**Health Status**	Good To Excellent Health	--*	--*
	Fair To Poor Health	1.063 (1.061, 1.064)	1.056 (1.054,1.059)
**Health Insurance Status**	Have Health Insurance	--*	--*
	Do Not Have Health Insurance	1.308 (1.306, 1.310)	1.174 (1.170,1.177)
**Health Care Provider**	Have HCP	--*	--*
	Do Not Have HCP	1.216 (1.215, 1.218)	1.146 (1.143,1.149)
**Deferment of Medical Care**	Medical Care Deferred Because Of Cost	1.136 (1.135, 1.138)	.903 (.901,.906)
	Medical Care Not Deferred	--*	--*
**Timing of Routine Medical Check Up**	Within Last 12 Months	--*	--*
	Longer Than 12 Months Ago	.992 (.991, .993)	1.125 (1.122,1.127)

*Referent Category.

The second logistic regression model also using low composite heart attack and stroke symptom knowledge score as the dependent variable and including only rural U.S. adults yielded similar results, many of which were magnified for rural Americans. Specifically, U.S. rural adults with low composite heart attack and stroke symptom knowledge scores were more likely to be male, >65 years of age, Hispanic and/or other/Multiracial, less educated (not a high school graduate or just a high school graduate), not married or living with a partner, living in a household with < $50,000 as an annual income, not have health insurance, not have a personal HCP, and have had a routine medical check-up longer than 12 months ago.

## Discussion

Our results suggest that U.S. adults living in rural locales have a knowledge deficit regarding heart attack and stroke symptomology as well as appropriate first response to heart attack and/or stroke. As with other research conducted on heart attack and stroke symptom knowledge in multiple groups (e.g., African American women, Hispanic males, African American men) [18-21], not all rural adults are likely to score low on knowledge questions about heart attack and stroke signs and symptoms. Our study identified the characteristics of rural adults who were more likely to do so. We found that rural adults who did not graduate from high school, those who were economically poorer or male or Hispanic were more likely to be low scorers regarding correct answers on heart attack and stroke symptomology questions.

For rural residents, knowing the signs and symptoms of heart attack and stoke is imperative given the importance of time from the onset of the event to appropriate treatment because of added distance to the hospital. While knowledge alone may be insufficient to spur action, it is the first step [26]. As such, tailored public health campaigns are essential for U.S. rural adults to enhance knowledge regarding heart attack and stroke symptomology.

We believe that pharmacists can and should play a role---perhaps even taking the lead---in helping to educate rural U.S. adults about the symptoms of heart attack and stroke. Community pharmacists, especially in rural communities, are easily accessible health care providers [26,27]. They may be the only health care providers in many rural communities. Pharmacist-driven public health related interventions have been successful in multiple communities for a variety of health concerns (e.g., smoking, hypertension, hyperlipidemia, elevated blood glucose) [28-32].

From the practice setting of a community pharmacy, pharmacists’ could mount low-cost campaigns to inform their rural patients about the signs and symptoms of heart attack and stroke. Elements of such a campaign may include: posters, medication counseling, pamphlets and community outreach programs. Furthermore, hospital-based pharmacists could partner with critical access hospitals to provided community outreach programs that not only provide education on the symptomology of strokes and heart attacks but also providing information of how to access care if and or when it might be needed. Finally, from the practice setting of primary care clinics, pharmacists could identify high-risk patients by using medical records and provide education during clinic visits. They might also offer group visits for high risk patients in order to provide additional education regarding CVD and heart attack and stroke signs and symptoms.

### Study limitations

Several potential limitations to this study should be noted. First, the survey is based on telephone derived data and may be skewed because those who could not be reached by phone could not participate in the survey. Additionally, widespread use of answering machines and caller identification now allow people to filter their phone calls potentially leading to a passive refusal to participate in surveys such as the BRFSS. However, call filtering is beyond the control of survey administrators. Furthermore, persons of lower socioeconomic status may have been excluded because of poorer phone access. However, the vast majority of U.S. residents live in households with telephones, which minimizes this bias. Moreover, U.S. cell phone numbers are now included in the pool of phones contacted for the survey. Nevertheless, study strength is in the use of a national database that included a robust sample of residents weighted to reflect the demographics of the U.S. population.

A second limitation is that the survey used close-ended questions, which limit participants’ options to fully explain response choices. Nonetheless, the survey questions were worded such that the answer choices covered a wide range of response possibilities. A third, and related, limitation is that the answers are self-reported, which introduces the possibility of recall bias on the part of the survey participants. A final potential bias resulted from the languages of the survey – English and Spanish. Individuals who did not speak English or Spanish were excluded from this survey.

## Conclusions

Analyses identified clear disparities between the knowledge levels of rural in comparison to non-rural U.S. adults regarding heart attack and stroke symptoms as well as appropriate first response to either event. These disparities should inform educational endeavors focused on improving knowledge of heart attack and stroke symptoms. Since pharmacists provide health care in multiple settings, they may be the best situated health care providers to take the lead in such public health education efforts.

## Misc

Michael T. Swanoski; M. Nawal Lutfiyya; Maria L. Amaro; Michael F. Akers; Krista L. Huot contributed equally.

## Competing interests

The authors declare that they have no competing interests.

## Authors’ contributions

MTS, MNL, MLA, MFA, KLH all made substantial contributions to conception and design of the manuscript, contributed to the interpretation of the data, were involved in revising the manuscript critically for important intellectual content, and have given final approval of this version of the manuscript to be published. Additionally, MNL oversaw: the completion of the first draft of the manuscript, the acquisition of the data and the statistical analyses. All authors read and approved the final manuscript.

## Pre-publication history

The pre-publication history for this paper can be accessed here:

http://www.biomedcentral.com/1471-2458/12/283/prepub
